# A prealbumin-containing TyG–FPR–AISI composite for clinically significant early sepsis-associated acute kidney injury: a single-center derivation cohort study with internal validation

**DOI:** 10.3389/fnut.2026.1825792

**Published:** 2026-07-27

**Authors:** Yang Gao, Chao Liu, Xinkui Xiong, Chao Yang, Sensheng Tang, Jiangyang Wan, Qiuni Chen, Luhao Jin, Lie Wang, Hui Xia, Zhiyun Xu

**Affiliations:** 1 Department of Cardiothoracic Surgery, The Affiliated Huai'an No. 1 People's Hospital of Nanjing Medical University, Huai'an, China; 2 Department of Urology, The Affiliated Huai'an No. 1 People's Hospital of Nanjing Medical University, Huai'an, China; 3 Department of Breast and Thyroid Surgery, The Affiliated Huai'an No. 1 People's Hospital of Nanjing Medical University, Huai'an, China; 4 Department of Hematology, The Affiliated Huai'an No. 1 People's Hospital of Nanjing Medical University, Huai'an, China; 5 Department of Neurosurgery, The Affiliated Huai'an No. 1 People's Hospital of Nanjing Medical University, Huai'an, China; 6 Department of Patient Relations, The Affiliated Huai'an No. 1 People's Hospital of Nanjing Medical University, Huai'an, China; 7 Department of Anesthesiology, The Affiliated Huai'an No. 1 People's Hospital of Nanjing Medical University, Huai'an, China

**Keywords:** aggregate index of systemic inflammation, catabolic-inflammatory status, fibrinogen-to-prealbumin ratio, host reserve, internal validation, risk stratification, sepsis-associated acute kidney injury, TyG index

## Abstract

**Background:**

Sepsis-associated acute kidney injury (SA-AKI) is heterogeneous in severity and biological drivers. Early identification of clinically significant SA-AKI remains challenging because conventional severity markers and kidney-function criteria do not fully capture metabolic stress, inflammatory-coagulopathy burden, and host-reserve/catabolic-inflammatory status. We evaluated whether a routinely available prealbumin-containing TyG–FPR–AISI composite provides incremental risk-stratification value for clinically significant early SA-AKI.

**Methods:**

This single-center retrospective derivation cohort included 359 septic ICU patients between January 2018 and December 2024. The primary endpoint was clinically significant early SA-AKI within 48 h after T0, defined as serum creatinine-defined KDIGO stage 2–3 AKI or renal-indication RRT/CRRT. Patients without the primary endpoint and with < 48 h of ICU observation were excluded from the main analysis, yielding 325 patients. TyG, fibrinogen-to-prealbumin ratio (FPR), and aggregate index of systemic inflammation (AISI) were measured within 0–24 h after T0. An equal-weight composite was calculated as the mean of standardized TyG, FPR, and AISI. Logistic regression, ROC analysis, fivefold cross-validation, calibration assessment, Brier score, decision curve analysis, and sensitivity analyses were performed.

**Results:**

Clinically significant early SA-AKI occurred in 69 of 325 patients (21.2%). FPR was independently associated with the primary endpoint (adjusted OR 1.36 per 1-SD increase, 95% CI 1.05–1.76), whereas TyG and AISI showed directionally positive but weaker associations. The equal-weight composite was independently associated with clinically significant early SA-AKI (adjusted OR 1.58 per 1-SD increase, 95% CI 1.20–2.07). Model 0 plus the composite improved apparent AUC from 0.671 to 0.706 and fivefold cross-validated AUC from 0.608 to 0.653, with a lower Brier score (0.147 vs. 0.155). Higher composite tertiles were associated with more severe KDIGO categories and persistent AKI at day 7. Sensitivity analyses supported the composite association for any-stage creatinine-defined SA-AKI, while associations were attenuated when urine-output criteria were incorporated.

**Conclusions:**

The prealbumin-containing TyG–FPR–AISI composite was associated with clinically significant early SA-AKI and short-term AKI persistence, but its incremental performance beyond a clinical baseline model was modest. These findings support its exploratory role as a routinely available nutrition-relevant host-response risk-enrichment signal rather than a stand-alone prediction tool; external validation and recalibration are required before clinical application.

## 1 Introduction

Sepsis-associated acute kidney injury (SA-AKI) is a frequent and clinically consequential complication among critically ill patients with sepsis. It is associated with prolonged ICU stay, increased requirement for renal replacement therapy (RRT), incomplete renal recovery, and higher mortality (1–3). Early identification of patients at risk remains challenging because SA-AKI is heterogeneous in timing, severity, and biological drivers. Moreover, not all early AKI events carry the same clinical implications. Mild KDIGO stage 1 creatinine changes may be more sensitive to baseline creatinine uncertainty, hemodilution, and measurement frequency in retrospective sepsis cohorts, whereas KDIGO stage 2–3 AKI and early renal-indication RRT/continuous renal replacement therapy (CRRT) represent more clinically consequential renal injury (1, 2, 4). Accordingly, risk stratification for clinically significant early SA-AKI may provide a relevant framework for identifying patients at risk of consequential kidney dysfunction during the early phase of sepsis.

The pathobiology of SA-AKI extends beyond renal hypoperfusion alone. Current evidence suggests that sepsis-related kidney injury reflects the interaction of systemic inflammation, endothelial and microcirculatory dysfunction, immune dysregulation, coagulation activation, metabolic stress, and impaired host adaptive capacity (4–7). These processes may converge to promote renal microvascular dysfunction, tubular stress, and persistent renal injury. However, the biological complexity of SA-AKI also limits the performance of single markers or severity scores when used in isolation. Conventional clinical indicators such as SOFA score, lactate, serum creatinine, and urine output are essential for assessing illness severity and diagnosing organ dysfunction, but they do not fully capture the multidimensional vulnerability state that may precede clinically significant SA-AKI. Kidney injury biomarkers such as NGAL, KIM-1, and IL-18 may provide more direct information on tubular injury, but they are not universally available in routine ICU practice and may be difficult to implement at scale (8, 9).

These limitations have encouraged interest in routinely available biomarker combinations that integrate complementary biological domains relevant to sepsis-related organ injury. Existing SA-AKI risk-stratification approaches rarely combine metabolic stress, inflammatory-coagulopathy burden, and host-reserve or catabolic-inflammatory signals within a single pragmatic framework (10, 11). Such an approach may be particularly useful in early sepsis, when risk assessment often depends on laboratory data already obtained during routine ICU care. Rather than replacing established clinical severity markers, a routinely available biomarker composite may provide incremental information beyond a clinical baseline model and help identify patients with a higher probability of clinically significant renal injury.

In this context, the triglyceride–glucose index (TyG), fibrinogen-to-prealbumin ratio (FPR), and aggregate index of systemic inflammation (AISI) represent three accessible indices aligned with distinct but potentially complementary host-response domains. TyG is a surrogate marker of stress-related metabolic dysregulation and insulin resistance, which have been linked to endothelial dysfunction and adverse outcomes in critical illness (12, 13). FPR integrates fibrinogen, an acute-phase reactant related to inflammation and coagulation activation, with prealbumin, a short half-life protein influenced by inflammation, hepatic synthesis, fluid shifts, and catabolic burden (14–18). Therefore, in sepsis, FPR should be interpreted as a prealbumin-containing coagulation–inflammation/host-reserve signal rather than as a pure nutritional-reserve marker. AISI combines neutrophil, monocyte, platelet, and lymphocyte counts and may reflect systemic inflammatory cell-count imbalance and immune-inflammatory activation (19, 20). These components were selected to represent complementary biological domains rather than because each component was expected to have equal prognostic strength. In a retrospective derivation setting, such a domain-based composite is useful only if it provides transparent and reproducible information beyond routine clinical severity variables (10, 11, 21–23).

We therefore developed and evaluated a TyG–FPR–AISI composite score for early risk stratification of clinically significant SA-AKI in septic ICU patients. To improve transparency and minimize overfitting, the primary composite was pre-specified as an equal-weight mean of standardized components; this design was not intended to imply equal biological importance or equal causal contribution of TyG, FPR, and AISI. A regression-weighted composite was evaluated as a sensitivity analysis to assess whether the findings depended on a particular weighting strategy. We hypothesized that the TyG–FPR–AISI composite would provide modest incremental, internally validated risk-stratification value beyond a clinical baseline model and would show a graded association with renal injury severity and short-term AKI persistence. Accordingly, the objectives of this study were to assess the associations of TyG, FPR, AISI, and their composite score with clinically significant early SA-AKI; to evaluate incremental model performance beyond a clinical baseline model using discrimination, calibration, internal validation, and decision curve analysis; and to examine the robustness of findings across alternative endpoint definitions, baseline creatinine strategies, AISI transformation, and composite-score constructions.

## 2 Methods

### 2.1 Study design and setting

This single-center retrospective derivation cohort study was conducted in the intensive care unit (ICU) of the Affiliated Huai'an No. 1 People's Hospital of Nanjing Medical University. Consecutive ICU admissions between January 2018 and December 2024 were screened. The study was designed and reported in accordance with the Strengthening the Reporting of Observational Studies in Epidemiology (STROBE) statement (24). The study protocol was approved by the Ethics Committee of Nanjing Medical University (Approval No.: KY-2026-073-01), and the requirement for written informed consent was waived because of the retrospective design and anonymized data processing. Because the study was conducted in a single-center retrospective cohort, all predictive analyses were considered exploratory and intended for derivation and internal validation only. The analytical framework included endpoint adjudication, predefined biomarker model development, assessment of discrimination and calibration, internal validation using cross-validation, and sensitivity analyses addressing baseline creatinine definition, urine-output criteria, AISI distribution, and composite-score construction (25–28).

### 2.2 Study population and patient selection

**Figure 1:**
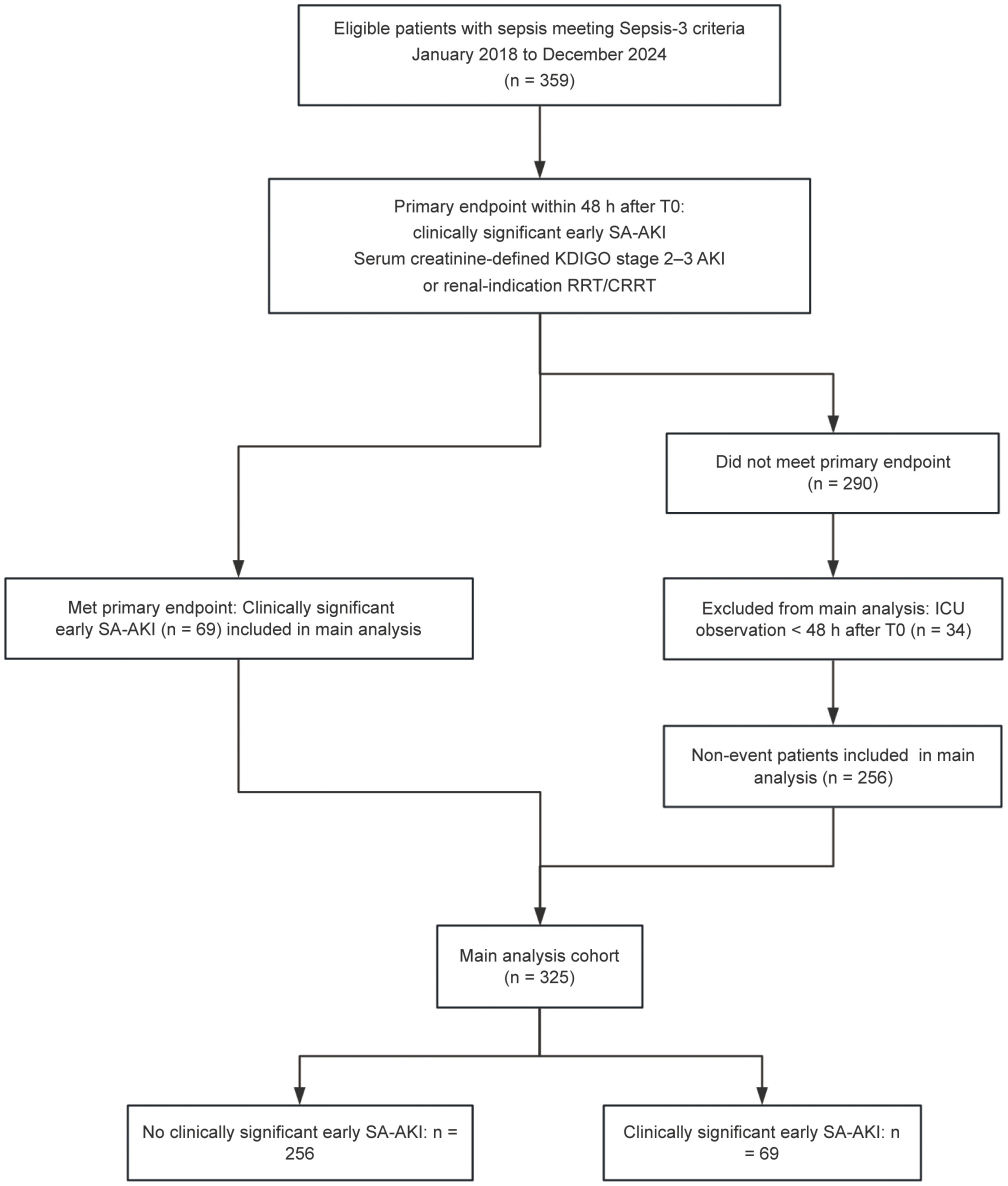
Flow diagram of patient selection and analytical cohorts. Eligible adult ICU patients with sepsis who met Sepsis-3 criteria between January 2018 and December 2024 were included in the eligible cohort (*n* = 359). T0 was defined as the first time point at which Sepsis-3 criteria were fulfilled; ICU admission time was used as a surrogate when the exact sepsis recognition time was unavailable. The primary endpoint was clinically significant early sepsis-associated acute kidney injury (SA-AKI), defined as serum creatinine-defined KDIGO stage 2–3 acute kidney injury or renal-indication renal replacement therapy/continuous renal replacement therapy (RRT/CRRT) within 48 h after T0. All patients who met the primary endpoint were retained in the main analysis (*n* = 69). Among patients who did not meet the primary endpoint (*n* = 290), those with less than 48 h of ICU observation after T0 were excluded from the main analysis (*n* = 34) to reduce outcome-ascertainment bias. The final main analysis cohort included 325 patients, comprising 69 patients with clinically significant early SA-AKI and 256 patients without clinically significant early SA-AKI.

Consecutive adult ICU patients were eligible if they were aged ≥18 years and met the Sepsis-3 definition during ICU stay, defined as suspected or confirmed infection plus an acute increase in Sequential Organ Failure Assessment (SOFA) score of ≥2 points from baseline (29). The time zero (T0) was defined as the first time point at which Sepsis-3 criteria were fulfilled. When the exact sepsis recognition time could not be reliably retrieved from the electronic medical record, ICU admission time was used as a surrogate T0. Patients were excluded if they had end-stage renal disease or chronic renal replacement therapy before admission, AKI already present before T0 or at ICU admission, pregnancy, hematologic malignancy or chemotherapy-related cytopenia, massive transfusion, missing laboratory data required to calculate TyG, FPR, or AISI within 0–24 h after T0, or missing data required for endpoint adjudication within the 0–48 h outcome window. For patients with multiple ICU admissions, only the first ICU admission was retained. A total of 359 patients constituted the eligible cohort. For the main analysis of clinically significant early SA-AKI, all patients who met the primary endpoint were included. Among patients who did not meet the primary endpoint, those with less than 48 h of ICU observation after T0 were excluded from the main analysis to reduce outcome-ascertainment bias. The final main analysis cohort included 325 patients. The patient selection and endpoint adjudication process is shown in Figure 1.

### 2.3 Endpoint definition and AKI adjudication

The primary endpoint was clinically significant early sepsis-associated acute kidney injury (SA-AKI) occurring within 48 h after T0. Clinically significant early SA-AKI was defined as serum creatinine-defined KDIGO stage 2–3 AKI or initiation of renal-indication renal replacement therapy (RRT) or continuous renal replacement therapy (CRRT) within 48 h after T0. RRT/CRRT was considered renal-indication when initiated for fluid overload, refractory acidosis, hyperkalemia, uremic complications, or mixed renal indications documented in the medical record. Serum creatinine-defined KDIGO stages were adjudicated according to KDIGO criteria as follows (30): KDIGO stage 1: increase in serum creatinine by ≥0.3 mg/dL within 48 h or increase to 1.5–1.9 times baseline; KDIGO stage 2: increase in serum creatinine to 2.0–2.9 times baseline; KDIGO stage 3: increase in serum creatinine to ≥3.0 times baseline, serum creatinine ≥4.0 mg/dL, or initiation of renal-indication RRT/CRRT within 48 h after T0. Baseline serum creatinine was defined using a hierarchical three-step approach to balance clinical plausibility and completeness in this retrospective sepsis cohort. First, when available, the most recent stable pre-admission serum creatinine measured within 7–365 days before ICU admission was used as the preferred baseline value. Second, for patients without available pre-admission creatinine, the first serum creatinine measured at hospital or ICU admission was used as the surrogate baseline. Third, when neither a reliable pre-admission value nor a clearly identifiable admission-first value was available, the lowest serum creatinine measured within 0–24 h after ICU admission was used as a fallback surrogate baseline. The source of baseline creatinine was recorded for each patient and compared between groups. To evaluate the potential influence of baseline creatinine assumptions on endpoint classification, pre-specified sensitivity analyses were performed using alternative baseline definitions, including restriction to patients with available pre-admission baseline creatinine, use of admission-first creatinine as baseline for all patients, and use of the lowest 0–24 h creatinine as a fallback baseline when pre-admission creatinine was unavailable. Any-stage creatinine-defined SA-AKI, defined as KDIGO stage 1–3 AKI or renal-indication RRT/CRRT within 48 h after T0, was analyzed as a key secondary endpoint. Because retrospective urine-output documentation was incomplete or partially charted in some patients, urine-output criteria were not included in the primary endpoint. A full KDIGO sensitivity analysis incorporating urine-output criteria was performed among patients with complete hourly urine-output records. The endpoint adjudication algorithm, missing-data structure, events-per-variable assessment, and multicollinearity diagnostics are summarized in Supplementary Table S1.

### 2.4 Biomarker measurements and calculations

All biomarker components were obtained within 0–24 h after T0, preferentially using the first available measurement closest to T0. The triglyceride–glucose index (TyG) was calculated as: TyG = ln [TG (mg/dL) × FPG (mg/dL)/2], where TG denotes triglycerides and FPG denotes fasting plasma glucose. If triglycerides or glucose were reported in mmol/L, unit conversion was performed before calculation: TG (mg/dL) = 88.57 × TG (mmol/L), FPG (mg/dL) = 18 × FPG (mmol/L). The fibrinogen-to-prealbumin ratio (FPR) was calculated as: FPR = fibrinogen/prealbumin, where fibrinogen and prealbumin were obtained within the same 0–24 h window and harmonized to consistent units according to local laboratory reporting. Because prealbumin in sepsis may be influenced by acute inflammation, hepatic synthesis, capillary leakage, fluid status, and catabolic-inflammatory burden, FPR was interpreted as a prealbumin-containing coagulation–inflammation/host-reserve signal rather than as a pure nutritional-reserve marker. The aggregate index of systemic inflammation (AISI) was calculated as: AISI = neutrophils × monocytes × platelets/lymphocytes, where neutrophils, monocytes, platelets, and lymphocytes denote absolute cell counts, typically expressed as × 10^9^/L.

### 2.5 Construction of the composite biomarker score

To enable comparability across biomarkers with different units and distributions, TyG, FPR, and AISI were standardized using z-transformation: Z(X) = [X – mean(X)]/SD(X), where mean(X) and SD(X) were calculated in the main analysis cohort. The primary composite score was constructed using an equal-weight approach: Composite_equal = [Z(TyG) + Z(FPR) + Z(AISI)]/3. This equal-weight strategy was pre-specified to give each host-response domain equal representation after standardization; it was not intended to imply that the three biomarkers have equal biological or causal importance. We selected the equal-weight score as the primary composite to improve transparency and reproducibility and to avoid deriving outcome-dependent weights from a single-center dataset with a limited number of events. A regression-weighted composite score was therefore evaluated only as a sensitivity analysis: Composite_weighted = 0.331 × Z(TyG) + 0.655 × Z(FPR) + 0.248 × Z(AISI). The weighted composite was standardized before effect-size reporting. Additional sensitivity analyses evaluated a composite score using log1p-transformed AISI to address the right-skewed distribution of AISI.

### 2.6 Covariates

Potential confounders were selected *a priori* based on clinical relevance and previous literature on sepsis-related kidney injury, while avoiding direct inclusion of outcome-defining components in the primary adjustment model. The baseline clinical adjustment model, referred to as Model 0, included age, sex, diabetes mellitus, chronic kidney disease, SOFA score within 0–24 h after T0, lactate within 0–24 h after T0, mechanical ventilation within 0–24 h after T0, and vasopressor use within 0–24 h after T0. All covariate data were collected by specially trained nurses and extracted from electronic medical records according to standardized definitions. Continuous variables included in regression models were standardized when effect sizes were reported per 1-SD increment.

### 2.7 Statistical analysis

Continuous variables were summarized as mean ± standard deviation or median with interquartile range, as appropriate. Categorical variables were summarized as counts and percentages. Between-group comparisons were performed using Welch's *t* test for approximately normally distributed continuous variables, the Mann–Whitney *U* test for skewed continuous variables, and the χ^2^ test or Fisher's exact test for categorical variables, as appropriate. Logistic regression was used to evaluate the associations of TyG, FPR, AISI, and composite scores with clinically significant early SA-AKI. Biomarkers and composite scores were modeled per 1-SD increment. Multivariable models were constructed by adding one biomarker or composite score to Model 0. The following predefined models were evaluated: Model 0; Model 0 + TyG; Model 0 + FPR; Model 0 + AISI; Model 0 + equal-weight composite; Model 0 + weighted composite. Adjusted odds ratios (ORs) were reported with 95% confidence intervals (CIs). The equal-weight composite was considered the primary composite model, whereas the weighted composite was analyzed as a sensitivity model. No data-driven variable selection, threshold optimization, or *post hoc* reweighting was used for the primary model. Model discrimination was assessed using receiver operating characteristic (ROC) curves and the area under the ROC curve (AUC). Apparent AUCs were calculated in the main analysis cohort. To evaluate internal reproducibility and potential optimism, stratified fivefold cross-validation was performed, and cross-validated AUCs were reported. Calibration was assessed using cross-validated calibration plots, calibration slope, calibration intercept, and Brier score (27). Absolute changes in AUC and Brier score relative to Model 0 were interpreted descriptively as measures of incremental performance rather than as evidence of clinical readiness. Clinical net benefit was evaluated by decision curve analysis across threshold probabilities from 0.05 to 0.50, comparing Model 0, Model 0 plus the composite score, treat-all, and treat-none strategies (28). To improve clinical interpretability, patients were categorized into tertiles of the equal-weight composite score. Event rates of clinically significant early SA-AKI, KDIGO severity distributions, and persistent AKI at day 7 were compared across composite-score tertiles. *P* values for trend were estimated by modeling composite-score tertile as an ordinal variable.

### 2.8 Sensitivity and secondary analyses

Several sensitivity analyses were conducted to evaluate the robustness of the findings. First, any-stage creatinine-defined SA-AKI, defined as KDIGO stage 1–3 AKI or renal-indication RRT/CRRT within 48 h after T0, was evaluated as a key secondary endpoint. Second, a full KDIGO sensitivity analysis incorporating urine-output criteria was performed among patients with complete hourly urine-output records. This analysis included serum creatinine criteria, urine-output criteria, and RRT/CRRT criteria. Third, baseline creatinine sensitivity analyses were performed to assess the robustness of endpoint classification to alternative baseline assumptions. These included: restriction to patients with available pre-admission baseline creatinine; use of first admission creatinine as baseline for all patients; and use of the lowest 0–24 h creatinine as the fallback baseline among patients without available pre-admission creatinine. Fourth, AISI-related sensitivity analyses were performed using log1p-transformed AISI to assess whether skewness or extreme values influenced model estimates. Fifth, composite-definition sensitivity analyses compared the equal-weight composite with the weighted composite and with a model including TyG, FPR, and AISI simultaneously. Sixth, secondary renal and clinical outcomes were explored, including persistent AKI at day 7, RRT/CRRT within 48 h, MAKE28, and renal recovery. These analyses were interpreted as exploratory and hypothesis-generating. Persistent AKI at day 7 was defined as ongoing KDIGO-defined AKI or continued RRT/CRRT requirement on day 7 after T0. Renal recovery was defined as resolution of AKI by day 7 without ongoing RRT/CRRT. MAKE28 was defined as a composite of death, new RRT/CRRT, or persistent renal dysfunction within 28 days after T0 (31). Events per variable were calculated to describe model complexity, and variance inflation factors were used to assess multicollinearity among clinical covariates and biomarker predictors (26). Missing-data patterns were summarized in Supplementary Table S1. Because key variables required for the primary models were complete in the main analysis cohort, the primary analyses were performed as complete-case analyses. All statistical tests were two-sided, and *P* < 0.05 was considered statistically significant. Analyses were performed using Python 3.12.11 (Python Software Foundation, Beaverton, OR, United States).

## 3 Results

### 3.1 Study population and baseline characteristics

Among 359 eligible septic ICU patients, 69 met the primary endpoint of clinically significant early SA-AKI within 48 h after T0. Among patients who did not meet the primary endpoint, 34 had less than 48 h of ICU observation after T0 and were excluded from the main analysis to reduce outcome-ascertainment bias. The final main analysis cohort included 325 patients, of whom 69 (21.2%) developed clinically significant early SA-AKI and 256 (78.8%) did not (Figure 1). Baseline, biomarker, and nutrition-related characteristics are summarized in Table 1. Compared with patients without clinically significant early SA-AKI, those with the endpoint had higher acute illness severity and greater hemodynamic instability, including a higher frequency of septic shock (56.5 vs. 32.4%, *P* < 0.001), higher SOFA scores within 0–24 h after T0 (7.07 ± 2.84 vs. 5.80 ± 2.62, *P* = 0.001), and more frequent vasopressor use (72.5 vs. 54.7%, *P* = 0.008). Pre-admission baseline creatinine was available in 212 patients (65.2%), with similar availability between groups (*P* = 0.571). Urine-output documentation quality was also comparable between groups (*P* = 0.907). Patients with clinically significant early SA-AKI had higher TyG index (9.51 ± 0.47 vs. 9.38 ± 0.56, *P* = 0.047), higher FPR [31.23 (21.63, 44.20) vs. 27.16 (19.99, 36.46), *P* = 0.042], and lower prealbumin levels (0.15 ± 0.07 vs. 0.17 ± 0.06 g/L, *P* = 0.032). AISI did not differ significantly in the unadjusted comparison [2,019.10 (850.40, 4,324.40) vs. 2,308.05 (1,103.27, 4,580.23), *P* = 0.755]. Nutrition-risk scores were higher in the endpoint group, including NRS2002 (3.81 ± 1.25 vs. 3.36 ± 1.33, *P* = 0.010) and mNUTRIC (4.32 ± 1.55 vs. 3.83 ± 1.72, *P* = 0.024), while early calorie and protein adequacy were lower.

**Table 1:** Baseline, biomarker, and nutrition-related characteristics according to clinically significant early SA-AKI.

Characteristic	Total (*n* = 325)	No clinically significant early SA-AKI (*n* = 256)	Clinically significant early SA-AKI (*n* = 69)	*P*-value
Demographics and major comorbidities				
Age, years	62.02 ± 15.69	61.30 ± 16.02	64.65 ± 14.20	0.094
Male sex	187 (57.5%)	148 (57.8%)	39 (56.5%)	0.847
BMI, kg/m^2^	24.22 ± 4.05	24.24 ± 4.08	24.17 ± 3.97	0.901
Diabetes mellitus	113 (34.8%)	83 (32.4%)	30 (43.5%)	0.087
Hypertension	159 (48.9%)	125 (48.8%)	34 (49.3%)	0.947
Chronic kidney disease	71 (21.8%)	57 (22.3%)	14 (20.3%)	0.724
Clinical severity and early ICU support				
Septic shock	122 (37.5%)	83 (32.4%)	39 (56.5%)	< 0.001
SOFA score (0–24 h)	6.07 ± 2.71	5.80 ± 2.62	7.07 ± 2.84	0.001
APACHE II score (0–24 h)	17.73 ± 5.51	17.48 ± 5.55	18.65 ± 5.31	0.110
Lactate (0–24 h), mmol/L	2.35 (1.73, 3.06)	2.25 (1.65, 3.00)	2.48 (1.86, 3.11)	0.086
Mechanical ventilation (0–24 h)	204 (62.8%)	155 (60.5%)	49 (71.0%)	0.110
Vasopressor use (0–24 h)	190 (58.5%)	140 (54.7%)	50 (72.5%)	0.008
MAP (0–24 h), mmHg	71.64 ± 11.86	71.31 ± 12.05	72.86 ± 11.11	0.316
Renal baseline and endpoint-adjudication variables				
Pre-admission baseline creatinine available	212 (65.2%)	165 (64.5%)	47 (68.1%)	0.571
Baseline creatinine source				0.830
Pre-admission 7–365 d	212 (65.2%)	165 (64.5%)	47 (68.1%)		
Admission first creatinine	59 (18.2%)	48 (18.8%)	11 (15.9%)		
Lowest creatinine within 0–24 h	54 (16.6%)	43 (16.8%)	11 (15.9%)		
Baseline creatinine used for endpoint, mg/dL	1.17 (0.85, 1.51)	1.21 (0.91, 1.52)	1.06 (0.63, 1.29)	< 0.001
Admission first creatinine, mg/dL	1.29 (0.99, 1.65)	1.25 (0.96, 1.63)	1.41 (1.15, 1.68)	0.050
Creatinine measurements within 0–48 h, count	3.00 (3.00, 4.00)	3.00 (2.00, 4.00)	6.00 (4.00, 6.00)	< 0.001
Urine-output documentation quality				0.907
Complete hourly charting	229 (70.5%)	179 (69.9%)	50 (72.5%)		
Partial nursing charting	82 (25.2%)	66 (25.8%)	16 (23.2%)		
Creatinine-only reliable	14 (4.3%)	11 (4.3%)	3 (4.3%)		
Core biomarkers measured within 0–24 h after T0				
TyG index	9.41 ± 0.54	9.38 ± 0.56	9.51 ± 0.47	0.047
FPR	28.10 (20.16, 37.48)	27.16 (19.99, 36.46)	31.23 (21.63, 44.20)	0.042
AISI	2,191.40 (1,006.40, 4,580.20)	2,308.05 (1,103.27, 4,580.23)	2,019.10 (850.40, 4,324.40)	0.755
Fibrinogen, g/L	4.39 ± 1.09	4.39 ± 1.08	4.38 ± 1.17	0.964
Prealbumin, g/L	0.16 ± 0.06	0.17 ± 0.06	0.15 ± 0.07	0.032
Albumin, g/L	29.30 ± 5.45	29.46 ± 5.42	28.72 ± 5.56	0.327
Nutrition-risk and early nutritional delivery variables				
Weight loss over 6 months, %	4.10 (2.60, 6.60)	4.20 (2.40, 6.82)	3.90 (3.10, 5.70)	0.779
NRS2002 score	3.46 ± 1.32	3.36 ± 1.33	3.81 ± 1.25	0.010
mNUTRIC score	3.93 ± 1.70	3.83 ± 1.72	4.32 ± 1.55	0.024
High nutrition risk	264 (81.2%)	204 (79.7%)	60 (87.0%)	0.170
Calorie adequacy within 0–48 h, %	57.71 ± 17.86	58.80 ± 17.56	53.67 ± 18.51	0.041
Protein adequacy within 0–48 h, %	52.57 ± 21.31	53.87 ± 21.64	47.78 ± 19.43	0.026

Data are presented as mean ± standard deviation, median (interquartile range), or number (percentage), as appropriate. The primary endpoint was clinically significant early SA-AKI, defined as serum creatinine-defined KDIGO stage 2–3 AKI or renal-indication RRT/CRRT within 48 h after T0. Continuous variables summarized as mean ± SD were compared using Welch's t test; skewed variables summarized as median (IQR) were compared using the Mann–Whitney U test; categorical variables were compared using the chi-square or Fisher's exact test, as appropriate. Secondary comorbidities (coronary artery disease, congestive heart failure, COPD, chronic liver disease, and malignancy), additional inflammatory markers (CRP and PCT), and detailed nutrition-intervention variables (reduced intake, enteral or parenteral nutrition, GI intolerance, and nutrition consultation) are provided in Supplementary Table S2.

### 3.2 Biomarker associations and model performance

**Figure 2:**
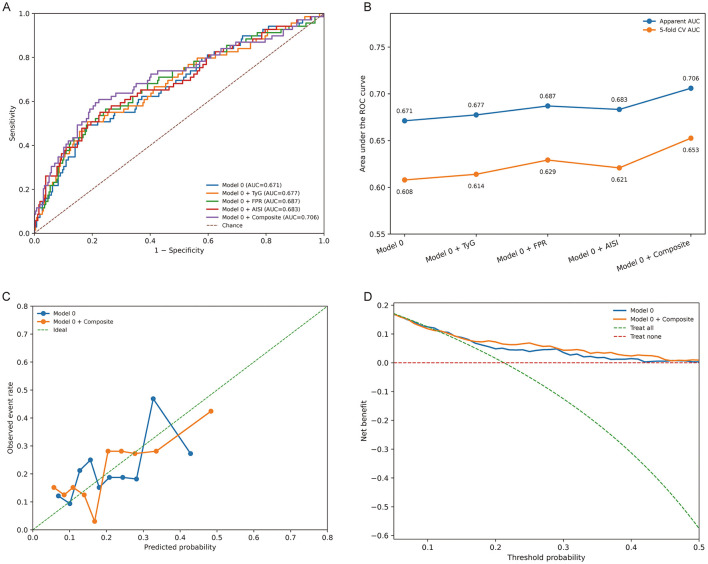
Discrimination, internal validation, calibration, and decision-curve assessment of biomarker-enhanced models for clinically significant early SA-AKI. Model performance was evaluated for the primary endpoint of clinically significant early sepsis-associated acute kidney injury (SA-AKI), defined as serum creatinine-defined KDIGO stage 2–3 acute kidney injury or renal-indication RRT/CRRT within 48 h after T0. Model 0 included age, sex, diabetes mellitus, chronic kidney disease, SOFA score, lactate, mechanical ventilation, and vasopressor use within 0–24 h after T0. Biomarker-enhanced models were constructed by adding TyG, fibrinogen-to-prealbumin ratio (FPR), aggregate index of systemic inflammation (AISI), or the equal-weight TyG–FPR–AISI composite to Model 0. The equal-weight composite was calculated as the mean of z-transformed TyG, FPR, and AISI. **(A)** Receiver operating characteristic curves showing apparent discrimination of Model 0 and biomarker-enhanced models in the main analysis cohort. **(B)** Comparison of apparent AUC and stratified fivefold cross-validated AUC across models. **(C)** Cross-validated calibration plot comparing predicted and observed event probabilities for Model 0 and Model 0 plus the equal-weight composite. The diagonal line represents ideal calibration. **(D)** Decision curve analysis comparing the net benefit of Model 0 and Model 0 plus the equal-weight composite across threshold probabilities from 0.05 to 0.50, with treat-all and treat-none strategies shown as references.

The associations of individual biomarkers and composite scores with clinically significant early SA-AKI are shown in Table 2. After adjustment for Model 0 covariates, FPR was independently associated with the primary endpoint (adjusted OR 1.36 per 1-SD increase, 95% CI 1.05–1.76, *P* = 0.018). TyG showed a positive but non-significant association (adjusted OR 1.24, 95% CI 0.93–1.66, *P* = 0.148), while AISI showed a borderline association (adjusted OR 1.27, 95% CI 0.99–1.63, *P* = 0.060). The equal-weight TyG–FPR–AISI composite was independently associated with clinically significant early SA-AKI (adjusted OR 1.58 per 1-SD increase, 95% CI 1.20–2.07, *P* = 0.001). The weighted composite showed similar results (adjusted OR 1.54, 95% CI 1.17–2.02, *P* = 0.002), indicating that the composite association was not dependent on a single weighting strategy. Model performance is summarized in Table 2 and Figure 2. Model 0 showed modest apparent discrimination (AUC 0.671), with a fivefold cross-validated AUC of 0.608. Adding the equal-weight composite increased the apparent AUC to 0.706 (absolute increase, 0.035) and the fivefold cross-validated AUC to 0.653 (absolute increase, 0.045). The Brier score decreased from 0.155 for Model 0 to 0.147 for Model 0 + equal-weight composite (absolute reduction, 0.008). Thus, the incremental performance of the composite was modest in magnitude. Cross-validated calibration showed a calibration slope/intercept of 0.68/−0.37 for Model 0 + equal-weight composite, compared with 0.53/−0.58 for Model 0. Decision curve analysis suggested greater net benefit for Model 0 + equal-weight composite than Model 0 across low-to-moderate threshold probabilities, although this decision-curve finding was interpreted as exploratory rather than as evidence of clinical implementation readiness.

**Table 2:** Primary associations and model performance for clinically significant early SA-AKI.

Model	Added predictor	Adjusted OR (95% CI)	*P*-value	Apparent AUC	Fivefold CV AUC	Brier score	CV calibration slope/intercept
Model 0	None; clinical baseline model	–	–	0.671	0.608	0.155	0.53/−0.58
Model 0 + TyG	TyG index, per 1-SD increase	1.24 (0.93–1.66)	0.148	0.677	0.614	0.153	0.57/−0.53
Model 0 + FPR	FPR, per 1-SD increase	1.36 (1.05–1.76)	0.018	0.687	0.629	0.151	0.63/−0.46
Model 0 + AISI	AISI, per 1-SD increase	1.27 (0.99–1.63)	0.060	0.683	0.621	0.152	0.55/−0.55
Model 0 + equal-weight composite	Equal-weight composite, per 1-SD increase	1.58 (1.20–2.07)	0.001	0.706	0.653	0.147	0.68/−0.37
Model 0 + weighted composite	Weighted composite, per 1-SD increase	1.54 (1.17–2.02)	0.002	0.704	0.653	0.148	0.68/−0.38

The primary endpoint was clinically significant early SA-AKI, defined as serum creatinine-defined KDIGO stage 2–3 AKI or renal-indication RRT/CRRT within 48 h after T0. Model 0 included age, sex, diabetes mellitus, chronic kidney disease, SOFA score, lactate, mechanical ventilation, and vasopressor use within 0–24 h after T0. Adjusted odds ratios for TyG, FPR, AISI, and composite scores are reported per 1-SD increment. The equal-weight composite was calculated as the mean of z(TyG), z(FPR), and z(AISI). The weighted composite was calculated as 0.331^*^z(TyG) + 0.655^*^z(FPR) + 0.248^*^z(AISI) and then standardized for effect-size reporting. Apparent AUC and Brier score were estimated in the full main analysis cohort; internally validated AUC and calibration metrics were estimated using fivefold cross-validation.

### 3.3 Composite-score risk gradient across renal severity and persistence

**Figure 3:**
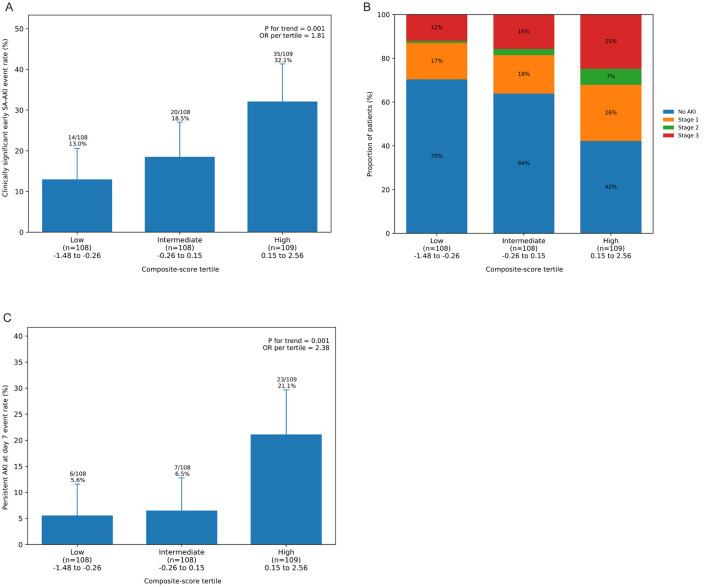
Risk gradients across tertiles of the equal-weight TyG–FPR–AISI composite score. Patients in the main analysis cohort were categorized into tertiles according to the equal-weight composite score, calculated as the mean of z-transformed TyG, fibrinogen-to-prealbumin ratio (FPR), and aggregate index of systemic inflammation (AISI). The tertile ranges are displayed below each group in the figure. The primary endpoint was clinically significant early sepsis-associated acute kidney injury (SA-AKI), defined as serum creatinine-defined KDIGO stage 2–3 acute kidney injury or renal-indication RRT/CRRT within 48 h after T0. **(A)** Event rates of clinically significant early SA-AKI across composite-score tertiles. The event rate increased from 13.0% in the low tertile to 18.5% in the intermediate tertile and 32.1% in the high tertile. **(B)** Distribution of serum creatinine- and RRT/CRRT-defined KDIGO AKI severity categories across composite-score tertiles. Higher composite-score tertiles showed a shift toward more severe AKI categories. **(C)** Event rates of persistent AKI at day 7 across composite-score tertiles. Persistent AKI at day 7 was defined as ongoing KDIGO-defined AKI or continued RRT/CRRT requirement on day 7 after T0. *P* values for trend were estimated by modeling composite-score tertile as an ordinal variable. Odds ratios per tertile represent the change in odds associated with each one-tertile increase in the composite-score category.

Patients were categorized into tertiles of the equal-weight composite score to evaluate clinical risk gradients (Figure 3). The incidence of clinically significant early SA-AKI increased across tertiles, from 13.0% in the low tertile to 18.5% in the intermediate tertile and 32.1% in the high tertile (*P* for trend < 0.001). The odds of clinically significant early SA-AKI increased by approximately 81% per tertile increment. The distribution of KDIGO severity categories also shifted across composite-score tertiles. The proportion of patients without AKI decreased from 70.4% in the low tertile to 42.2% in the high tertile, whereas KDIGO stage 3 increased from 12.0 to 24.8%. Persistent AKI at day 7 similarly increased across tertiles, from 5.6% in the low tertile to 6.5% in the intermediate tertile and 21.1% in the high tertile (*P* for trend < 0.001).

### 3.4 Secondary and sensitivity analyses

Secondary and sensitivity analyses are summarized in Table 3. For the key secondary endpoint of any-stage creatinine-defined SA-AKI, the equal-weight composite remained independently associated with the outcome (adjusted OR 1.57, 95% CI 1.22–2.01, *P* < 0.001), although discrimination was modest (AUC 0.645). In the full KDIGO sensitivity analysis restricted to patients with complete hourly urine-output records, the association was attenuated after incorporating urine-output criteria (adjusted OR 1.13, 95% CI 0.83–1.54, *P* = 0.423). Baseline creatinine sensitivity analyses were generally supportive of the main findings. The composite remained associated with the primary endpoint among patients with available pre-admission baseline creatinine (adjusted OR 1.72, 95% CI 1.18–2.50, *P* = 0.005) and when the lowest 0–24 h creatinine was used as a fallback baseline (adjusted OR 1.57, 95% CI 1.20–2.07, *P* = 0.001), whereas the estimate was attenuated when admission first creatinine was used as baseline for all patients (adjusted OR 1.31, 95% CI 0.97–1.76, *P* = 0.079). The association also persisted when log1p-transformed AISI was used in the composite and when the weighted composite was analyzed. For secondary outcomes, the composite was associated with persistent AKI at day 7 (adjusted OR 1.72, 95% CI 1.25–2.38, *P* < 0.001). In contrast, associations with RRT/CRRT within 48 h and MAKE28 were weaker and not statistically significant, suggesting that the composite was more closely related to clinically significant renal injury and short-term AKI persistence than to treatment-decision-dependent RRT initiation or broader composite outcomes.

**Table 3:** Secondary and sensitivity analyses.

Analysis	Endpoint/cohort	*N*	Events	Adjusted OR for composite (95% CI)	*P*-value	AUC	Key interpretation
Key secondary endpoint	Any-stage creatinine-defined SA-AKI (KDIGO stage 1–3 or renal-indication RRT/CRRT within 48 h)	333	142	1.57 (1.22–2.01)	< 0.001	0.645	Composite remained associated with any-stage SA-AKI, although discrimination was modest
Full KDIGO sensitivity	Any-stage SA-AKI using serum creatinine, urine-output criteria, or RRT/CRRT among complete hourly urine-output records	243	176	1.13 (0.83–1.54)	0.423	0.633	Association attenuated when urine-output criteria were included, consistent with greater noise in retrospective urine-output adjudication
Baseline Scr sensitivity	Primary endpoint restricted to patients with available pre-admission baseline creatinine	212	47	1.72 (1.18–2.50)	0.005	0.778	Association was preserved when analysis was restricted to patients with pre-admission baseline creatinine
Baseline Scr sensitivity	Primary endpoint using admission first creatinine as baseline for all patients	324	51	1.31 (0.97–1.76)	0.079	0.708	Effect estimate was attenuated using admission first creatinine for all patients
Baseline Scr sensitivity	Primary endpoint using lowest 0–24 h creatinine as fallback when pre-admission creatinine was unavailable	325	70	1.57 (1.20–2.07)	0.001	0.711	Results were similar to the main analysis when the previous fallback baseline approach was used
AISI sensitivity	Primary endpoint using composite with log1p(AISI) instead of raw AISI	325	69	1.37 (1.03–1.83)	0.029	0.689	Association persisted after using log-transformed AISI, supporting robustness to AISI skewness
Composite-definition sensitivity	Primary endpoint using weighted TyG–FPR–AISI composite	325	69	1.54 (1.17–2.02)	0.002	0.704	Weighted and equal-weight composites showed comparable performance
Internal-validation sensitivity	Primary endpoint using equal-weight composite with fivefold cross-validated predictions	325	69	1.58 (1.20–2.07)	0.001	0.706; CV 0.653	Discrimination was reduced after cross-validation but remained above the clinical baseline model
Secondary renal outcome	Persistent AKI at day 7 in the main analysis cohort	325	36	1.72 (1.25–2.38)	< 0.001	0.780	Composite was associated with persistent AKI at day 7
Secondary renal outcome	RRT/CRRT within 48 h in the main analysis cohort	325	34	0.88 (0.59–1.31)	0.533	0.757	Composite was not independently associated with RRT/CRRT, which may reflect treatment-decision heterogeneity
Secondary composite outcome	MAKE28 in the main analysis cohort	325	93	1.18 (0.92–1.51)	0.200	0.674	Association with MAKE28 was weaker, supporting a kidney-injury rather than broad outcome-prediction interpretation

Unless otherwise specified, all analyses used Model 0 plus the equal-weight composite score, with adjustment for age, sex, diabetes mellitus, chronic kidney disease, SOFA score, lactate, mechanical ventilation, and vasopressor use. The equal-weight composite was calculated as the mean of z(TyG), z(FPR), and z(AISI), using the main analysis cohort as the reference distribution. Adjusted odds ratios are reported per 1-SD increment in the composite score. AUC values are apparent AUCs unless a cross-validated AUC is explicitly shown. The primary endpoint was clinically significant early SA-AKI, defined as serum creatinine-defined KDIGO stage 2–3 AKI or renal-indication RRT/CRRT within 48 h after T0. Full KDIGO sensitivity analysis included urine-output criteria only among patients with complete hourly urine-output records.

### 3.5 Distributional assessment and model diagnostics

AISI showed a right-skewed distribution, supporting the use of log1p(AISI) in sensitivity analysis (Supplementary Figure S1). Spearman correlation analysis showed limited overlap among TyG, FPR, and AISI, supporting their interpretation as partially distinct host-response signals rather than redundant measures. FPR was strongly inversely correlated with prealbumin and moderately correlated with fibrinogen, as expected from its formula, whereas TyG and AISI showed weak correlations with SOFA score, lactate, and APACHE II score. Missing-data patterns, events-per-variable estimates, and multicollinearity diagnostics are summarized in Supplementary Table S1. Key variables required for the primary models were complete in the main analysis cohort. The events-per-variable ratio was 8.6 for Model 0 and 7.7 for Model 0 + equal-weight composite, indicating a limited but interpretable number of primary endpoint events relative to model complexity. Variance inflation factors were low for Model 0 + equal-weight composite and for the simultaneous biomarker model, indicating no concerning multicollinearity.

## 4 Discussion

In this single-center retrospective derivation cohort of septic ICU patients, we evaluated routinely available biomarkers representing metabolic stress, inflammatory-coagulopathy burden, and systemic inflammatory cell-count imbalance for early risk stratification of clinically significant SA-AKI. Using a primary endpoint focused on KDIGO stage 2–3 AKI or renal-indication RRT/CRRT within 48 h after T0, the equal-weight TyG–FPR–AISI composite was independently associated with clinically significant early SA-AKI after adjustment for a clinical baseline model. The composite also demonstrated a graded association with renal injury severity and persistent AKI at day 7. However, its incremental discrimination beyond Model 0 was modest, with an apparent AUC of 0.706 and a fivefold cross-validated AUC of 0.653. Therefore, these findings should be interpreted as hypothesis-generating evidence for a candidate risk-enrichment signal rather than as validation of a stand-alone clinical prediction tool.

The clinical interpretation of the composite should be cautious. The absolute increase in cross-validated AUC after adding the equal-weight composite to Model 0 was 0.045, and the absolute reduction in Brier score was 0.008. These changes suggest incremental information but not a decisive improvement in prediction. In addition, the cross-validated AUC was lower than the apparent AUC, and the calibration slope below 1 indicated residual optimism, as expected in an internally validated single-center derivation model. Thus, the composite may complement established clinical assessment based on illness severity, hemodynamics, kidney function, and clinician judgment, but it should not replace these elements. External validation, recalibration, and assessment of clinical impact are required before any bedside use can be considered.

The biological rationale for integrating TyG, FPR, and AISI lies in the multidimensional nature of sepsis-related kidney injury and in the partial distinction among their host-response domains. TyG reflects stress-related metabolic dysregulation and insulin-resistance-like physiology; FPR integrates an acute-phase coagulation marker with a short half-life negative acute-phase protein; and AISI captures systemic inflammatory cell-count imbalance. These domains are aligned with mechanisms implicated in SA-AKI, including metabolic stress, endothelial dysfunction, inflammatory-coagulopathy, immune dysregulation, and microcirculatory injury (4–7, 32–35). The equal-weight approach was selected to provide transparent domain representation and to reduce dependence on outcome-derived weights from a limited single-center dataset; it should not be interpreted as evidence that the three components have equal biological importance. The similar findings obtained with the weighted composite support that the overall association was not driven solely by one arbitrary weighting strategy. However, the present study was observational and did not directly measure endothelial injury, microcirculatory function, glycocalyx disruption, mitochondrial dysfunction, or immune phenotypes. Therefore, our findings should be interpreted as being consistent with, rather than proving, these mechanistic pathways. This distinction is important because the composite was developed for pragmatic risk stratification, not for mechanistic phenotyping.

Among the individual biomarkers, FPR showed the clearest independent association with clinically significant early SA-AKI, whereas TyG and AISI showed directionally positive but weaker associations after adjustment. The stronger performance of FPR may reflect the combined influence of coagulation-inflammation burden and prealbumin-related host response. However, prealbumin should not be interpreted as a pure nutritional-reserve marker in sepsis. It is strongly influenced by acute inflammation, hepatic synthesis, capillary leakage, fluid balance, and catabolic stress (15–18, 36). Accordingly, the FPR component of the composite is better understood as a prealbumin-containing coagulation–inflammation/host-reserve or catabolic-inflammatory signal rather than as a direct measure of nutritional status. Although patients with clinically significant early SA-AKI had higher NRS2002 and mNUTRIC scores and lower early calorie and protein adequacy, this study did not test nutritional interventions or establish that modifying nutritional delivery changes SA-AKI risk (18, 36–38). The findings therefore should not be used to infer that the composite can guide nutrition therapy without prospective evaluation (16, 17, 36).

The sensitivity analyses provide important context for interpreting the main findings. The composite remained associated with any-stage creatinine-defined SA-AKI, although discrimination was modest, suggesting that the signal extends beyond severe AKI but is less specific for milder stage 1 events. In contrast, when urine-output criteria were incorporated among patients with complete hourly urine-output records, the association was attenuated. This may reflect the greater heterogeneity and potential noise of retrospective urine-output adjudication, particularly in critically ill patients with variable fluid administration, diuretic use, catheter documentation, and nursing charting patterns (2, 30). Baseline creatinine sensitivity analyses were generally supportive: the association persisted in patients with available pre-admission creatinine and when the lowest 0–24 h creatinine was used as a fallback, whereas it was attenuated when admission first creatinine was applied to all patients. Together, these findings support the decision to focus the primary endpoint on creatinine-defined clinically significant early SA-AKI while retaining any-stage and full KDIGO definitions as sensitivity analyses.

The risk-gradient analyses further strengthen the clinical interpretability of the composite. Patients in the highest composite-score tertile had a higher incidence of clinically significant early SA-AKI, a shift toward more severe KDIGO categories, and a higher rate of persistent AKI at day 7 (31). This pattern suggests that the composite may be more closely related to renal injury severity and short-term persistence than to broad clinical outcomes. Consistent with this interpretation, associations with RRT/CRRT initiation and MAKE28 were weaker and not statistically significant. RRT/CRRT initiation is partly dependent on clinician decision-making, local practice patterns, fluid strategy, electrolyte disturbances, and resource availability, whereas MAKE28 incorporates multiple non-biomarker determinants (2, 7, 31, 39). Therefore, the composite should not be interpreted as a general outcome-prediction model for all kidney-related or mortality-related endpoints.

This study has several strengths. First, the exposure window for biomarker measurement and the outcome window for early renal injury were explicitly defined, preserving temporal interpretability. Second, the primary endpoint focused on clinically significant early SA-AKI, while any-stage SA-AKI and full KDIGO definitions were retained as secondary or sensitivity analyses. Third, the composite score was evaluated against a clinical baseline model and assessed using discrimination, calibration, internal validation, Brier score, decision curve analysis, and risk-gradient plots. Fourth, the use of an equal-weight composite as the primary score improved transparency and reduced reliance on regression-derived weights from a single dataset, while the weighted composite was retained for comparison. Finally, the biomarkers used in the composite are routinely available in many ICU settings, supporting feasibility for future external validation studies.

Several limitations should also be acknowledged. First and most importantly, this was a single-center retrospective derivation study, and external validation and recalibration are mandatory before the composite can be considered for clinical use. Second, although internal validation was performed using fivefold cross-validation, the calibration slope below 1 and the difference between apparent and cross-validated performance indicate residual optimism (25, 27, 40). Third, the number of primary endpoint events was limited relative to model complexity; although events-per-variable diagnostics and multicollinearity assessments did not show major instability, overfitting cannot be excluded. Fourth, baseline creatinine uncertainty remains a potential source of misclassification despite sensitivity analyses. Fifth, urine-output criteria were not included in the primary endpoint because retrospective urine-output documentation was incomplete or partially charted in some patients; although a full KDIGO sensitivity analysis was performed, urine-output-based adjudication remains vulnerable to documentation heterogeneity. Sixth, the equal-weight composite was designed as a pragmatic and transparent domain-based score rather than a biologically derived weighting system. Seventh, the study did not measure mechanistic intermediates such as endothelial markers, microcirculatory parameters, glycocalyx injury, or immune phenotypes, limiting causal and mechanistic inference. Finally, the study did not evaluate whether interventions guided by the composite score improve renal outcomes.

In summary, the TyG–FPR–AISI composite was associated with clinically significant early SA-AKI and showed modest incremental risk-stratification value beyond a clinical baseline model. Its association with KDIGO severity distribution and persistent AKI suggests potential utility as a risk-enrichment signal for clinically meaningful renal injury in early sepsis. However, because this was a single-center retrospective derivation study with internal validation only, the composite should be considered exploratory until externally validated and recalibrated in independent cohorts.

## Data Availability

The original contributions presented in the study are included in the article/Supplementary material, further inquiries can be directed to the corresponding authors.

## Supplementary material

Supplementary Figure S1 Distributional assessment of AISI and correlation structure among biomarkers, severity indicators, and nutrition-related variables. (A) Histogram of the raw aggregate index of systemic inflammation (AISI), showing a right-skewed distribution with high-value outliers. (B) Histogram of log1p-transformed AISI, demonstrating improved distributional symmetry after transformation. The dashed vertical lines indicate the median values. (C) Boxplot of raw AISI according to clinically significant early SA-AKI status, illustrating the distribution and extreme values of AISI in patients with and without the primary endpoint. (D) Spearman rank correlation matrix among TyG, fibrinogen-to-prealbumin ratio (FPR), AISI, SOFA score, lactate, APACHE II score, prealbumin, fibrinogen, NRS2002 score, and mNUTRIC score. Cell values represent Spearman correlation coefficients. The heatmap was used to assess overlap among candidate biomarkers, clinical severity indicators, and nutrition-related variables.

Supplementary Table S1 Endpoint adjudication algorithm, missing data, events-per-variable, and multicollinearity diagnostics.

Supplementary Table S2 Full baseline characteristics and full model coefficients.
