# Supplementary material for: A prealbumin-containing TyG–FPR–AISI composite for clinically significant early sepsis-associated acute kidney injury: a single-center derivation cohort study with internal validation
Source: Front Nutr. 2026 Jul 27;13:1825792. doi: 10.3389/fnut.2026.1825792 (PMC13453680; doi:10.3389/fnut.2026.1825792)
Supplement: Supplementary file 1 [file Table_1.DOCX]

Table S1. Endpoint adjudication algorithm, missing data, events-per-variable, and multicollinearity diagnostics

Part A. Endpoint adjudication algorithm

| Domain | Rule | Use |
| --- | --- | --- |
| T0 | First Sepsis-3 recognition; ICU admission surrogate if exact time unavailable. | All analyses |
| Primary endpoint | KDIGO stage 2–3 by serum creatinine or renal-indication RRT/CRRT within 48 h after T0. | Main analysis |
| Key secondary endpoint | Any-stage KDIGO stage 1–3 by serum creatinine or renal-indication RRT/CRRT within 48 h. | Secondary analysis |
| Baseline Scr | Pre-admission Scr within 7–365 d if available; otherwise admission-first Scr; if neither was reliably available, lowest Scr within 0–24 h was used as fallback. | Main analysis |
| KDIGO stage 1 | Scr increase ≥0.3 mg/dL within 48 h or 1.5–1.9 times baseline. | Secondary endpoint only |
| KDIGO stage 2 | Scr 2.0–2.9 times baseline. | Primary endpoint component |
| KDIGO stage 3 | Scr ≥3.0 times baseline, Scr ≥4.0 mg/dL, or RRT/CRRT within 48 h. | Primary endpoint component |
| Urine output | Excluded from primary endpoint; assessed only in complete hourly urine-output records. | Sensitivity analysis |
| Main analysis set | All primary-endpoint events plus non-events with ≥48 h ICU observation (n=325, events=69). | Main analysis |
| Baseline sensitivity | Pre-admission Scr subgroup; admission-first Scr for all; lowest 0–24 h Scr fallback. | Table 3 |

Part B. Missing data in the main analysis cohort

| Variable group | Maximum missing n | Maximum missing % | Details |
| --- | --- | --- | --- |
| Primary endpoint components | 0 | 0.0% | prior_creatinine_available: 0 (0.0%); baseline_creatinine_mg_dL: 0 (0.0%); admission_first_creatinine_mg_dL: 0 (0.0%); peak_creatinine_0_48h_mg_dL: 0 (0.0%); RRT_indication: 0 (0.0%) |
| Model 0 covariates | 0 | 0.0% | Age_years: 0 (0.0%); Sex: 0 (0.0%); Diabetes: 0 (0.0%); CKD: 0 (0.0%); SOFA_0_24h: 0 (0.0%); Lactate_mmol_L_0_24h: 0 (0.0%); Mechanical_ventilation_0_24h: 0 (0.0%); Vasopressor_0_24h: 0 (0.0%) |
| Core biomarkers | 0 | 0.0% | TyG_index: 0 (0.0%); FPR: 0 (0.0%); AISI: 0 (0.0%) |
| Nutrition variables | 0 | 0.0% | NRS2002_score: 0 (0.0%); mNUTRIC_score: 0 (0.0%); high_nutrition_risk: 0 (0.0%); calorie_adequacy_0_48h_pct: 0 (0.0%); protein_adequacy_0_48h_pct: 0 (0.0%) |
| Urine-output data | 0 | 0.0% | urine_output_documentation_quality: 0 (0.0%); urine_output_mL_kg_h_0_6h: 0 (0.0%); oliguria_6h: 0 (0.0%) |
| Secondary outcomes | 0 | 0.0% | persistent_AKI_day7: 0 (0.0%); renal_recovery_by_day7: 0 (0.0%); MAKE28: 0 (0.0%); Mortality_28d: 0 (0.0%); In_hospital_mortality: 0 (0.0%) |
| RRT start time | 290 | NA | Missing by design for patients without RRT; not treated as missing. |

Part C. Events-per-variable and model complexity

| Analysis | N | Events | Model | Predictors | EPV |
| --- | --- | --- | --- | --- | --- |
| Primary endpoint | 325 | 69 | Model 0 | 8 | 8.6 |
| Primary endpoint | 325 | 69 | Model 0 + single biomarker | 9 | 7.7 |
| Primary endpoint | 325 | 69 | Model 0 + composite | 9 | 7.7 |
| Primary endpoint | 325 | 69 | Model 0 + TyG + FPR + AISI | 11 | 6.3 |
| Any-stage SA-AKI | 333 | 142 | Model 0 + composite | 9 | 15.8 |
| Pre-admission Scr subgroup | 212 | 47 | Model 0 + composite | 9 | 5.2 |

Part D. Multicollinearity diagnostics

| Model | Variable | VIF |
| --- | --- | --- |
| Model 0 + composite | Age | 1.03 |
| Model 0 + composite | Male sex | 1.90 |
| Model 0 + composite | Diabetes | 1.38 |
| Model 0 + composite | CKD | 1.21 |
| Model 0 + composite | SOFA | 1.18 |
| Model 0 + composite | Lactate | 1.06 |
| Model 0 + composite | Mechanical ventilation | 2.19 |
| Model 0 + composite | Vasopressor use | 2.13 |
| Model 0 + composite | Composite | 1.05 |
| Model 0 + TyG + FPR + AISI | TyG | 1.05 |
| Model 0 + TyG + FPR + AISI | FPR | 1.07 |
| Model 0 + TyG + FPR + AISI | AISI | 1.04 |

Footnote. VIF values were calculated in the main analysis cohort after standardizing continuous variables. A VIF <5 was considered to indicate no concerning multicollinearity. RRT_start_hours_after_T0 was missing by design in patients without RRT and was not treated as an endpoint missingness variable.
