# Supplementary material for: A prealbumin-containing TyG–FPR–AISI composite for clinically significant early sepsis-associated acute kidney injury: a single-center derivation cohort study with internal validation
Source: Front Nutr. 2026 Jul 27;13:1825792. doi: 10.3389/fnut.2026.1825792 (PMC13453680; doi:10.3389/fnut.2026.1825792)
Supplement: Supplementary file 2 [file Table_2.DOCX]

Table S2. Full baseline characteristics and full model coefficients

Part A1. Demographics, admission characteristics, and comorbidities

| Characteristic | Total (n=325) | No clinically significant early SA-AKI (n=256) | Clinically significant early SA-AKI (n=69) | P value |
| --- | --- | --- | --- | --- |
| Demographics, admission characteristics, and comorbidities |  |  |  |  |
| Age, years | 62.02 ± 15.69 | 61.30 ± 16.02 | 64.65 ± 14.20 | 0.094 |
| BMI, kg/m² | 24.22 ± 4.05 | 24.24 ± 4.08 | 24.17 ± 3.97 | 0.901 |
| Hospital days before ICU | 2.00 (1.00, 4.00) | 2.00 (1.00, 4.00) | 2.00 (1.00, 3.00) | 0.022 |
| Male sex | 187 (57.5%) | 148 (57.8%) | 39 (56.5%) | 0.847 |
| Admission source |  |  |  | 0.654 |
| ED | 164 (50.5%) | 126 (49.2%) | 38 (55.1%) |  |
| OR/PACU | 38 (11.7%) | 29 (11.3%) | 9 (13.0%) |  |
| Transfer | 40 (12.3%) | 34 (13.3%) | 6 (8.7%) |  |
| Ward | 83 (25.5%) | 67 (26.2%) | 16 (23.2%) |  |
| Infection site |  |  |  | 0.958 |
| Pulmonary | 119 (36.6%) | 93 (36.3%) | 26 (37.7%) |  |
| Abdominal | 80 (24.6%) | 61 (23.8%) | 19 (27.5%) |  |
| Urinary | 42 (12.9%) | 34 (13.3%) | 8 (11.6%) |  |
| Bloodstream | 40 (12.3%) | 33 (12.9%) | 7 (10.1%) |  |
| Skin/Soft tissue | 18 (5.5%) | 15 (5.9%) | 3 (4.3%) |  |
| Other | 26 (8.0%) | 20 (7.8%) | 6 (8.7%) |  |
| T0 source |  |  |  | 0.188 |
| Exact sepsis recognition time | 244 (75.1%) | 188 (73.4%) | 56 (81.2%) |  |
| ICU admission surrogate | 81 (24.9%) | 68 (26.6%) | 13 (18.8%) |  |
| Diabetes mellitus | 113 (34.8%) | 83 (32.4%) | 30 (43.5%) | 0.087 |
| Hypertension | 159 (48.9%) | 125 (48.8%) | 34 (49.3%) | 0.947 |
| Chronic kidney disease | 71 (21.8%) | 57 (22.3%) | 14 (20.3%) | 0.724 |
| Coronary artery disease | 60 (18.5%) | 48 (18.8%) | 12 (17.4%) | 0.796 |
| Congestive heart failure | 35 (10.8%) | 30 (11.7%) | 5 (7.2%) | 0.287 |
| COPD | 48 (14.8%) | 37 (14.5%) | 11 (15.9%) | 0.757 |
| Chronic liver disease | 38 (11.7%) | 32 (12.5%) | 6 (8.7%) | 0.383 |
| Malignancy | 34 (10.5%) | 30 (11.7%) | 4 (5.8%) | 0.187 |

Part A2. Clinical severity, vital signs, and early ICU support

| Characteristic | Total (n=325) | No clinically significant early SA-AKI (n=256) | Clinically significant early SA-AKI (n=69) | P value |
| --- | --- | --- | --- | --- |
| Clinical severity, vital signs, and early ICU support |  |  |  |  |
| Septic shock | 122 (37.5%) | 83 (32.4%) | 39 (56.5%) | <0.001 |
| Mechanical ventilation (0–24 h) | 204 (62.8%) | 155 (60.5%) | 49 (71.0%) | 0.110 |
| Vasopressor use (0–24 h) | 190 (58.5%) | 140 (54.7%) | 50 (72.5%) | 0.008 |
| SOFA score (0–24 h) | 6.07 ± 2.71 | 5.80 ± 2.62 | 7.07 ± 2.84 | 0.001 |
| APACHE II score (0–24 h) | 17.73 ± 5.51 | 17.48 ± 5.55 | 18.65 ± 5.31 | 0.110 |
| Lactate (0–24 h), mmol/L | 2.35 (1.73, 3.06) | 2.25 (1.65, 3.00) | 2.48 (1.86, 3.11) | 0.086 |
| MAP (0–24 h), mmHg | 71.64 ± 11.86 | 71.31 ± 12.05 | 72.86 ± 11.11 | 0.316 |
| Heart rate (0–24 h), bpm | 103.54 ± 17.39 | 102.64 ± 17.23 | 106.86 ± 17.73 | 0.081 |
| Respiratory rate (0–24 h), /min | 24.21 ± 5.75 | 24.18 ± 5.74 | 24.30 ± 5.81 | 0.878 |
| Temperature (0–24 h), °C | 37.77 ± 0.80 | 37.74 ± 0.80 | 37.87 ± 0.80 | 0.237 |
| SpO₂ (0–24 h), % | 93.86 ± 3.75 | 93.93 ± 3.82 | 93.59 ± 3.48 | 0.493 |

Part A3. Renal baseline, urine-output records, and kidney outcomes

| Characteristic | Total (n=325) | No clinically significant early SA-AKI (n=256) | Clinically significant early SA-AKI (n=69) | P value |
| --- | --- | --- | --- | --- |
| Renal baseline, urine-output records, and kidney outcomes |  |  |  |  |
| Pre-admission baseline creatinine available | 212 (65.2%) | 165 (64.5%) | 47 (68.1%) | 0.571 |
| Baseline creatinine source |  |  |  | 0.830 |
| Pre-admission 7-365d | 212 (65.2%) | 165 (64.5%) | 47 (68.1%) |  |
| Admission first creatinine | 59 (18.2%) | 48 (18.8%) | 11 (15.9%) |  |
| Lowest creatinine within 0-24h | 54 (16.6%) | 43 (16.8%) | 11 (15.9%) |  |
| Urine-output documentation quality |  |  |  | 0.907 |
| Complete hourly charting | 229 (70.5%) | 179 (69.9%) | 50 (72.5%) |  |
| Partial nursing charting | 82 (25.2%) | 66 (25.8%) | 16 (23.2%) |  |
| Creatinine-only reliable | 14 (4.3%) | 11 (4.3%) | 3 (4.3%) |  |
| Baseline creatinine used for endpoint, mg/dL | 1.17 (0.85, 1.51) | 1.21 (0.91, 1.52) | 1.06 (0.63, 1.29) | <0.001 |
| Baseline eGFR, mL/min/1.73 m² | 71.33 ± 27.70 | 68.37 ± 26.45 | 82.33 ± 29.61 | <0.001 |
| Admission first creatinine, mg/dL | 1.29 (0.99, 1.65) | 1.25 (0.96, 1.63) | 1.41 (1.15, 1.68) | 0.050 |
| Lowest creatinine within 0–24 h, mg/dL | 1.17 (0.90, 1.50) | 1.11 (0.86, 1.50) | 1.23 (1.05, 1.50) | 0.105 |
| Creatinine at 24–48 h, mg/dL | 1.34 (0.99, 1.73) | 1.21 (0.93, 1.62) | 1.70 (1.35, 2.19) | <0.001 |
| Peak creatinine within 0–48 h, mg/dL | 1.47 (1.14, 1.98) | 1.38 (1.10, 1.80) | 2.20 (1.49, 3.00) | <0.001 |
| Creatinine measurements within 0–48 h, count | 3.00 (3.00, 4.00) | 3.00 (2.00, 4.00) | 6.00 (4.00, 6.00) | <0.001 |
| Urine output 0–24 h, mL | 814.11 ± 548.66 | 863.46 ± 546.26 | 631.00 ± 521.66 | 0.002 |
| Urine output 0–6 h, mL/kg/h | 0.56 ± 0.54 | 0.60 ± 0.55 | 0.42 ± 0.51 | 0.011 |
| ICU length of stay, days | 7.00 (5.00, 10.00) | 7.00 (5.00, 10.00) | 9.00 (6.00, 12.00) | 0.008 |
| Hospital length of stay, days | 18.00 (13.00, 24.00) | 17.00 (12.00, 22.00) | 23.00 (16.00, 27.00) | <0.001 |
| Oliguria within 0–6 h | 189 (58.2%) | 139 (54.3%) | 50 (72.5%) | 0.007 |
| RRT/CRRT within 48 h | 34 (10.5%) | 0 (0.0%) | 34 (49.3%) | <0.001 |
| Persistent AKI at day 7 | 36 (11.1%) | 6 (2.3%) | 30 (43.5%) | <0.001 |
| Renal recovery by day 7 | 0 (0.0%) | 0 (0.0%) | 0 (0.0%) | 1.000 |
| MAKE28 | 93 (28.6%) | 33 (12.9%) | 60 (87.0%) | <0.001 |
| 28-day mortality | 41 (12.6%) | 27 (10.5%) | 14 (20.3%) | 0.031 |
| In-hospital mortality | 28 (8.6%) | 18 (7.0%) | 10 (14.5%) | 0.050 |

Part A4. Routine biomarkers, inflammatory, coagulation, and kidney-injury markers

| Characteristic | Total (n=325) | No clinically significant early SA-AKI (n=256) | Clinically significant early SA-AKI (n=69) | P value |
| --- | --- | --- | --- | --- |
| Routine biomarkers, inflammatory, coagulation, and kidney-injury markers |  |  |  |  |
| Fasting plasma glucose, mg/dL | 148.72 ± 43.46 | 146.28 ± 44.21 | 157.77 ± 39.55 | 0.039 |
| Triglycerides, mg/dL | 187.35 ± 70.01 | 186.79 ± 71.86 | 189.45 ± 63.12 | 0.764 |
| TyG index | 9.41 ± 0.54 | 9.38 ± 0.56 | 9.51 ± 0.47 | 0.047 |
| Fibrinogen, g/L | 4.39 ± 1.09 | 4.39 ± 1.08 | 4.38 ± 1.17 | 0.964 |
| Prealbumin, g/L | 0.16 ± 0.06 | 0.17 ± 0.06 | 0.15 ± 0.07 | 0.032 |
| FPR | 28.10 (20.16, 37.48) | 27.16 (19.99, 36.46) | 31.23 (21.63, 44.20) | 0.042 |
| WBC, ×10⁹/L | 16.67 ± 6.22 | 16.70 ± 6.23 | 16.58 ± 6.22 | 0.888 |
| Neutrophils, ×10⁹/L | 14.18 ± 5.98 | 14.16 ± 5.92 | 14.26 ± 6.22 | 0.905 |
| Lymphocytes, ×10⁹/L | 0.90 ± 0.46 | 0.91 ± 0.46 | 0.84 ± 0.48 | 0.268 |
| Monocytes, ×10⁹/L | 0.89 ± 0.36 | 0.88 ± 0.36 | 0.91 ± 0.38 | 0.539 |
| Platelets, ×10⁹/L | 185.38 ± 90.13 | 192.50 ± 92.52 | 159.00 ± 75.53 | 0.002 |
| Hemoglobin, g/L | 101.37 ± 20.48 | 101.79 ± 21.05 | 99.81 ± 18.31 | 0.442 |
| AISI | 2191.40 (1006.40, 4580.20) | 2308.05 (1103.27, 4580.23) | 2019.10 (850.40, 4324.40) | 0.755 |
| CRP, mg/L | 116.27 ± 58.29 | 119.06 ± 59.56 | 105.92 ± 52.43 | 0.076 |
| PCT, ng/mL | 1.41 (0.77, 2.32) | 1.45 (0.78, 2.39) | 1.31 (0.74, 2.02) | 0.452 |
| Albumin, g/L | 29.30 ± 5.45 | 29.46 ± 5.42 | 28.72 ± 5.56 | 0.327 |
| Total bilirubin, µmol/L | 19.30 (12.71, 29.58) | 20.25 (13.50, 29.46) | 16.40 (8.41, 31.10) | 0.025 |
| ALT, U/L | 33.00 (23.00, 49.00) | 34.00 (23.00, 49.00) | 30.00 (22.00, 49.00) | 0.772 |
| AST, U/L | 41.00 (27.00, 63.00) | 41.00 (27.75, 64.25) | 38.00 (26.00, 54.00) | 0.260 |
| BUN, mmol/L | 7.10 (5.40, 9.30) | 6.90 (5.10, 8.70) | 8.90 (6.13, 10.61) | <0.001 |
| Cystatin C, mg/L | 1.44 (1.22, 1.61) | 1.43 (1.20, 1.61) | 1.46 (1.25, 1.60) | 0.218 |
| D-dimer, mg/L FEU | 2.41 (1.52, 4.07) | 2.37 (1.53, 4.28) | 2.72 (1.48, 3.81) | 0.845 |
| INR | 1.16 (1.08, 1.26) | 1.16 (1.07, 1.26) | 1.18 (1.08, 1.26) | 0.373 |
| APTT, sec | 37.48 ± 6.42 | 36.98 ± 6.53 | 39.33 ± 5.68 | 0.004 |
| DIC score | 2.00 (1.00, 3.00) | 2.00 (1.00, 2.00) | 2.00 (2.00, 3.00) | <0.001 |
| NGAL, ng/mL | 142.00 (103.00, 215.00) | 133.00 (95.00, 179.00) | 293.00 (141.00, 474.00) | <0.001 |
| KIM-1, pg/mL | 506.00 (335.00, 728.00) | 452.00 (310.75, 619.75) | 894.00 (573.00, 1254.00) | <0.001 |
| IL-18, pg/mL | 112.00 (75.00, 186.00) | 100.00 (71.75, 152.00) | 249.00 (145.00, 387.00) | <0.001 |

Part A5. Fluid balance, nephrotoxin exposure, infection management, and nutrition

| Characteristic | Total (n=325) | No clinically significant early SA-AKI (n=256) | Clinically significant early SA-AKI (n=69) | P value |
| --- | --- | --- | --- | --- |
| Fluid balance, nephrotoxin exposure, infection management, and nutrition |  |  |  |  |
| Fluid input 0–24 h, mL | 3370.96 ± 714.83 | 3328.77 ± 727.96 | 3527.46 ± 644.90 | 0.029 |
| Fluid balance 0–24 h, mL | 1732.25 ± 1014.98 | 1642.64 ± 1017.53 | 2064.72 ± 939.92 | 0.002 |
| Cumulative fluid balance 0–48 h, mL | 2646.05 ± 1423.74 | 2479.12 ± 1373.43 | 3265.38 ± 1445.86 | <0.001 |
| Maximum norepinephrine, µg/kg/min | 0.07 (0.00, 0.15) | 0.05 (0.00, 0.13) | 0.12 (0.00, 0.21) | 0.001 |
| Vasopressor hours 0–48 h | 16.60 (0.00, 31.30) | 14.10 (0.00, 28.52) | 28.70 (0.00, 43.30) | <0.001 |
| Time to antibiotics, h | 1.90 (1.30, 2.80) | 1.90 (1.40, 2.83) | 1.70 (1.20, 2.40) | 0.168 |
| Weight loss over 6 months, % | 4.10 (2.60, 6.60) | 4.20 (2.40, 6.82) | 3.90 (3.10, 5.70) | 0.779 |
| NRS2002 score | 3.46 ± 1.32 | 3.36 ± 1.33 | 3.81 ± 1.25 | 0.010 |
| mNUTRIC score | 3.93 ± 1.70 | 3.83 ± 1.72 | 4.32 ± 1.55 | 0.024 |
| Energy target, kcal/day | 1633.70 ± 306.66 | 1631.59 ± 296.12 | 1641.52 ± 345.21 | 0.828 |
| Calories delivered 0–48 h, kcal/day | 943.77 ± 350.66 | 963.62 ± 352.44 | 870.09 ± 336.30 | 0.045 |
| Calorie adequacy 0–48 h, % | 57.71 ± 17.86 | 58.80 ± 17.56 | 53.67 ± 18.51 | 0.041 |
| Protein target, g/day | 81.65 ± 15.33 | 81.54 ± 14.81 | 82.03 ± 17.25 | 0.831 |
| Protein delivered 0–48 h, g/day | 43.06 ± 19.60 | 44.20 ± 20.03 | 38.83 ± 17.39 | 0.029 |
| Protein adequacy 0–48 h, % | 52.57 ± 21.31 | 53.87 ± 21.64 | 47.78 ± 19.43 | 0.026 |
| Diuretic use 0–48 h | 55 (16.9%) | 37 (14.5%) | 18 (26.1%) | 0.022 |
| Nephrotoxin exposure 0–48 h | 190 (58.5%) | 153 (59.8%) | 37 (53.6%) | 0.358 |
| Vancomycin 0–48 h | 110 (33.8%) | 88 (34.4%) | 22 (31.9%) | 0.698 |
| Aminoglycoside 0–48 h | 16 (4.9%) | 15 (5.9%) | 1 (1.4%) | 0.209 |
| IV contrast 0–72 h | 43 (13.2%) | 34 (13.3%) | 9 (13.0%) | 0.959 |
| Recent NSAID or ACEI/ARB | 66 (20.3%) | 49 (19.1%) | 17 (24.6%) | 0.314 |
| Appropriate antibiotics within 24 h | 286 (88.0%) | 221 (86.3%) | 65 (94.2%) | 0.094 |
| Source control within 48 h | 0 (0.0%) | 0 (0.0%) | 0 (0.0%) | 1.000 |
| Reduced intake within 7 days | 0 (0.0%) | 0 (0.0%) | 0 (0.0%) | 1.000 |
| High nutrition risk | 264 (81.2%) | 204 (79.7%) | 60 (87.0%) | 0.170 |
| Enteral nutrition started within 48 h | 171 (52.6%) | 135 (52.7%) | 36 (52.2%) | 0.934 |
| Parenteral nutrition within 0–72 h | 70 (21.5%) | 59 (23.0%) | 11 (15.9%) | 0.203 |
| GI intolerance 0–48 h | 68 (20.9%) | 53 (20.7%) | 15 (21.7%) | 0.851 |
| Nutrition consultation within 48 h | 146 (44.9%) | 107 (41.8%) | 39 (56.5%) | 0.029 |
| Data quality flag |  |  |  | 0.464 |
| Complete key variables | 255 (78.5%) | 199 (77.7%) | 56 (81.2%) |  |
| Minor timing uncertainty | 46 (14.2%) | 39 (15.2%) | 7 (10.1%) |  |
| Baseline Scr uncertainty | 21 (6.5%) | 15 (5.9%) | 6 (8.7%) |  |
| Urine output incomplete | 3 (0.9%) | 3 (1.2%) | 0 (0.0%) |  |

Part B1. Full model coefficients: Model 0

| Model | Variable | β coefficient | SE | OR | 95% CI | P value |
| --- | --- | --- | --- | --- | --- | --- |
| Model 0 | Age, per 1-SD increase | 0.133 | 0.144 | 1.14 | 0.86-1.51 | 0.358 |
| Model 0 | Male sex | 0.101 | 0.286 | 1.11 | 0.63-1.94 | 0.723 |
| Model 0 | Diabetes mellitus | 0.483 | 0.289 | 1.62 | 0.92-2.85 | 0.094 |
| Model 0 | Chronic kidney disease | -0.121 | 0.349 | 0.89 | 0.45-1.76 | 0.729 |
| Model 0 | SOFA score, per 1-SD increase | 0.377 | 0.163 | 1.46 | 1.06-2.01 | 0.021 |
| Model 0 | Lactate, per 1-SD increase | 0.148 | 0.135 | 1.16 | 0.89-1.51 | 0.271 |
| Model 0 | Mechanical ventilation | 0.186 | 0.315 | 1.20 | 0.65-2.23 | 0.554 |
| Model 0 | Vasopressor use | 0.497 | 0.321 | 1.64 | 0.88-3.08 | 0.121 |

Part B2. Full model coefficients: Model 0 + TyG

| Model | Variable | β coefficient | SE | OR | 95% CI | P value |
| --- | --- | --- | --- | --- | --- | --- |
| Model 0 + TyG | Age, per 1-SD increase | 0.131 | 0.146 | 1.14 | 0.86-1.52 | 0.367 |
| Model 0 + TyG | Male sex | 0.060 | 0.288 | 1.06 | 0.60-1.87 | 0.836 |
| Model 0 + TyG | Diabetes mellitus | 0.390 | 0.297 | 1.48 | 0.83-2.64 | 0.189 |
| Model 0 + TyG | Chronic kidney disease | -0.096 | 0.351 | 0.91 | 0.46-1.81 | 0.784 |
| Model 0 + TyG | SOFA score, per 1-SD increase | 0.397 | 0.165 | 1.49 | 1.08-2.05 | 0.016 |
| Model 0 + TyG | Lactate, per 1-SD increase | 0.144 | 0.136 | 1.15 | 0.88-1.51 | 0.290 |
| Model 0 + TyG | Mechanical ventilation | 0.169 | 0.317 | 1.18 | 0.64-2.20 | 0.593 |
| Model 0 + TyG | Vasopressor use | 0.458 | 0.323 | 1.58 | 0.84-2.97 | 0.156 |
| Model 0 + TyG | TyG index, per 1-SD increase | 0.216 | 0.149 | 1.24 | 0.93-1.66 | 0.148 |

Part B3. Full model coefficients: Model 0 + FPR

| Model | Variable | β coefficient | SE | OR | 95% CI | P value |
| --- | --- | --- | --- | --- | --- | --- |
| Model 0 + FPR | Age, per 1-SD increase | 0.114 | 0.146 | 1.12 | 0.84-1.49 | 0.434 |
| Model 0 + FPR | Male sex | 0.089 | 0.289 | 1.09 | 0.62-1.93 | 0.759 |
| Model 0 + FPR | Diabetes mellitus | 0.475 | 0.292 | 1.61 | 0.91-2.85 | 0.104 |
| Model 0 + FPR | Chronic kidney disease | -0.077 | 0.350 | 0.93 | 0.47-1.84 | 0.827 |
| Model 0 + FPR | SOFA score, per 1-SD increase | 0.399 | 0.166 | 1.49 | 1.08-2.06 | 0.016 |
| Model 0 + FPR | Lactate, per 1-SD increase | 0.105 | 0.139 | 1.11 | 0.85-1.46 | 0.450 |
| Model 0 + FPR | Mechanical ventilation | 0.118 | 0.319 | 1.12 | 0.60-2.10 | 0.712 |
| Model 0 + FPR | Vasopressor use | 0.337 | 0.329 | 1.40 | 0.73-2.67 | 0.306 |
| Model 0 + FPR | FPR, per 1-SD increase | 0.307 | 0.130 | 1.36 | 1.05-1.76 | 0.018 |

Part B4. Full model coefficients: Model 0 + AISI

| Model | Variable | β coefficient | SE | OR | 95% CI | P value |
| --- | --- | --- | --- | --- | --- | --- |
| Model 0 + AISI | Age, per 1-SD increase | 0.171 | 0.147 | 1.19 | 0.89-1.58 | 0.245 |
| Model 0 + AISI | Male sex | 0.097 | 0.287 | 1.10 | 0.63-1.93 | 0.735 |
| Model 0 + AISI | Diabetes mellitus | 0.506 | 0.291 | 1.66 | 0.94-2.93 | 0.082 |
| Model 0 + AISI | Chronic kidney disease | -0.218 | 0.358 | 0.80 | 0.40-1.62 | 0.542 |
| Model 0 + AISI | SOFA score, per 1-SD increase | 0.403 | 0.166 | 1.50 | 1.08-2.07 | 0.015 |
| Model 0 + AISI | Lactate, per 1-SD increase | 0.121 | 0.137 | 1.13 | 0.86-1.48 | 0.374 |
| Model 0 + AISI | Mechanical ventilation | 0.144 | 0.317 | 1.15 | 0.62-2.15 | 0.650 |
| Model 0 + AISI | Vasopressor use | 0.489 | 0.322 | 1.63 | 0.87-3.07 | 0.129 |
| Model 0 + AISI | AISI, per 1-SD increase | 0.238 | 0.126 | 1.27 | 0.99-1.63 | 0.060 |

Part B5. Full model coefficients: Model 0 + equal-weight composite

| Model | Variable | β coefficient | SE | OR | 95% CI | P value |
| --- | --- | --- | --- | --- | --- | --- |
| Model 0 + equal-weight composite | Age, per 1-SD increase | 0.156 | 0.149 | 1.17 | 0.87-1.57 | 0.296 |
| Model 0 + equal-weight composite | Male sex | 0.038 | 0.291 | 1.04 | 0.59-1.84 | 0.896 |
| Model 0 + equal-weight composite | Diabetes mellitus | 0.394 | 0.296 | 1.48 | 0.83-2.65 | 0.183 |
| Model 0 + equal-weight composite | Chronic kidney disease | -0.161 | 0.358 | 0.85 | 0.42-1.72 | 0.652 |
| Model 0 + equal-weight composite | SOFA score, per 1-SD increase | 0.444 | 0.169 | 1.56 | 1.12-2.17 | 0.009 |
| Model 0 + equal-weight composite | Lactate, per 1-SD increase | 0.080 | 0.141 | 1.08 | 0.82-1.43 | 0.572 |
| Model 0 + equal-weight composite | Mechanical ventilation | 0.072 | 0.322 | 1.07 | 0.57-2.02 | 0.824 |
| Model 0 + equal-weight composite | Vasopressor use | 0.312 | 0.330 | 1.37 | 0.72-2.61 | 0.344 |
| Model 0 + equal-weight composite | Equal-weight composite, per 1-SD increase | 0.455 | 0.139 | 1.58 | 1.20-2.07 | 0.001 |

Part B6. Full model coefficients: Model 0 + weighted composite

| Model | Variable | β coefficient | SE | OR | 95% CI | P value |
| --- | --- | --- | --- | --- | --- | --- |
| Model 0 + weighted composite | Age, per 1-SD increase | 0.129 | 0.148 | 1.14 | 0.85-1.52 | 0.383 |
| Model 0 + weighted composite | Male sex | 0.047 | 0.291 | 1.05 | 0.59-1.85 | 0.873 |
| Model 0 + weighted composite | Diabetes mellitus | 0.413 | 0.295 | 1.51 | 0.85-2.70 | 0.162 |
| Model 0 + weighted composite | Chronic kidney disease | -0.105 | 0.353 | 0.90 | 0.45-1.80 | 0.766 |
| Model 0 + weighted composite | SOFA score, per 1-SD increase | 0.430 | 0.168 | 1.54 | 1.11-2.14 | 0.011 |
| Model 0 + weighted composite | Lactate, per 1-SD increase | 0.081 | 0.141 | 1.08 | 0.82-1.43 | 0.566 |
| Model 0 + weighted composite | Mechanical ventilation | 0.072 | 0.321 | 1.07 | 0.57-2.02 | 0.822 |
| Model 0 + weighted composite | Vasopressor use | 0.273 | 0.331 | 1.31 | 0.69-2.52 | 0.410 |
| Model 0 + weighted composite | Weighted composite, per 1-SD increase | 0.431 | 0.138 | 1.54 | 1.17-2.02 | 0.002 |

Part B7. Full model coefficients: Model 0 + TyG + FPR + AISI

| Model | Variable | β coefficient | SE | OR | 95% CI | P value |
| --- | --- | --- | --- | --- | --- | --- |
| Model 0 + TyG + FPR + AISI | Age, per 1-SD increase | 0.150 | 0.151 | 1.16 | 0.86-1.56 | 0.321 |
| Model 0 + TyG + FPR + AISI | Male sex | 0.041 | 0.292 | 1.04 | 0.59-1.85 | 0.888 |
| Model 0 + TyG + FPR + AISI | Diabetes mellitus | 0.408 | 0.302 | 1.50 | 0.83-2.72 | 0.177 |
| Model 0 + TyG + FPR + AISI | Chronic kidney disease | -0.152 | 0.362 | 0.86 | 0.42-1.75 | 0.674 |
| Model 0 + TyG + FPR + AISI | SOFA score, per 1-SD increase | 0.441 | 0.169 | 1.55 | 1.12-2.17 | 0.009 |
| Model 0 + TyG + FPR + AISI | Lactate, per 1-SD increase | 0.076 | 0.142 | 1.08 | 0.82-1.42 | 0.592 |
| Model 0 + TyG + FPR + AISI | Mechanical ventilation | 0.066 | 0.322 | 1.07 | 0.57-2.01 | 0.838 |
| Model 0 + TyG + FPR + AISI | Vasopressor use | 0.293 | 0.333 | 1.34 | 0.70-2.58 | 0.379 |
| Model 0 + TyG + FPR + AISI | TyG index, per 1-SD increase | 0.216 | 0.153 | 1.24 | 0.92-1.68 | 0.159 |
| Model 0 + TyG + FPR + AISI | FPR, per 1-SD increase | 0.307 | 0.130 | 1.36 | 1.05-1.75 | 0.018 |
| Model 0 + TyG + FPR + AISI | AISI, per 1-SD increase | 0.237 | 0.127 | 1.27 | 0.99-1.63 | 0.062 |

Footnote. Part A data are presented as mean ± standard deviation, median (interquartile range), or number (percentage), as appropriate. Continuous variables summarized as mean ± SD were compared using Welch's t test; skewed variables summarized as median (IQR) were compared using the Mann-Whitney U test; categorical variables were compared using the chi-square or Fisher's exact test, as appropriate. Part B reports logistic regression coefficients for the primary endpoint, clinically significant early SA-AKI. Continuous variables in the models were standardized and modeled per 1-SD increment. Model 0 included age, sex, diabetes mellitus, chronic kidney disease, SOFA score, lactate, mechanical ventilation, and vasopressor use.
